# Supplementary material for: Optimizing the Power to Identify the Genetic Basis of Complex Traits with Evolve and Resequence Studies
Source: Mol Biol Evol. 2019 Aug 10;36(12):2890–905. doi: 10.1093/molbev/msz183 (PMC6878953; doi:10.1093/molbev/msz183)
Supplement: msz183_Supplementary_Data [file msz183_supplementary_data.zip › Vlachos_Kofler_suppl.pdf]

Supplement to Optimizing the power to identify the genetic basis of complex traits with Evolve and Resequence studies

Christos Vlachos and Robert Kofler

July 18, 2019

1 Supplementary figures

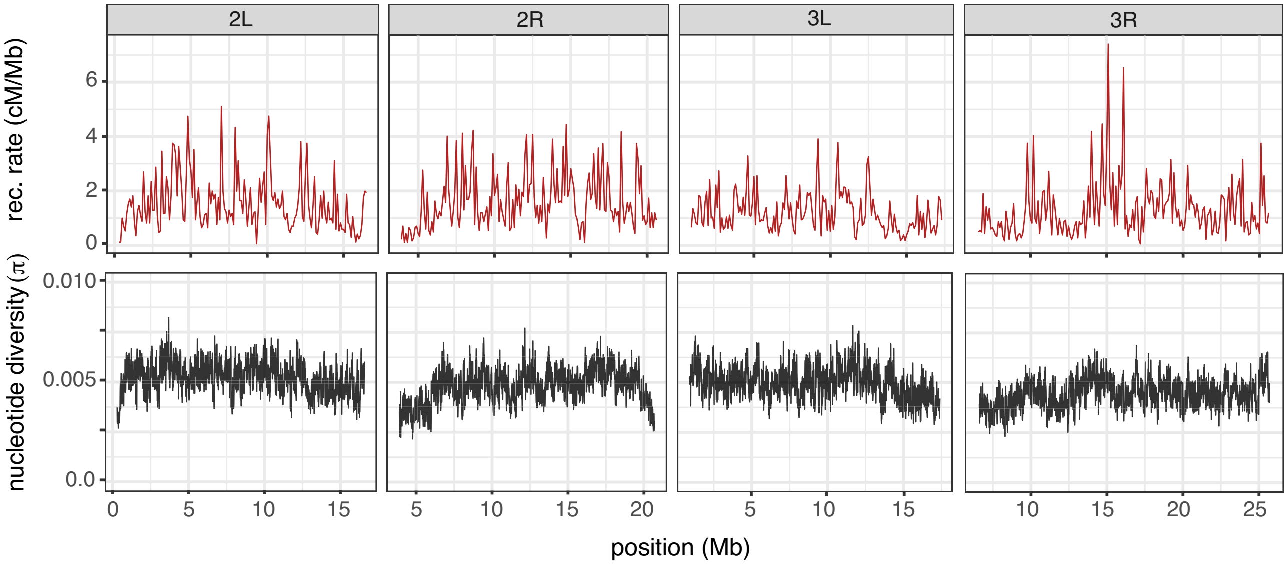

Figure 1: The genomic landscape used in the simulations. The recombination rate is from Comeron et al. (2012) and the base population captures the nucleotide diversity of a natural *D. melanogaster* population from Vienna (Bastide et al., 2013). The window size size is 100kb.

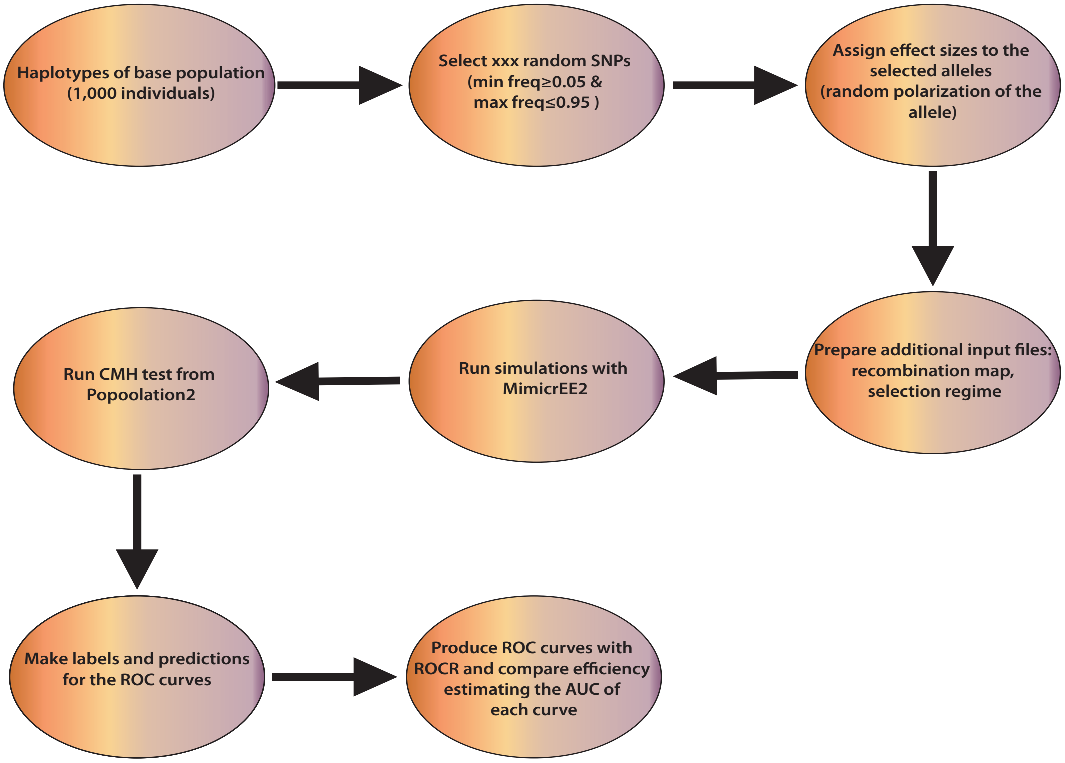

Figure 2: Overview of our simulation pipeline. The best selection regime was identified based on the largest Area Under the Curve (AUC) of each Receiver Operating Characteristic (ROC) plot.

1.1 Allele frequency changes in truncation selection simulations

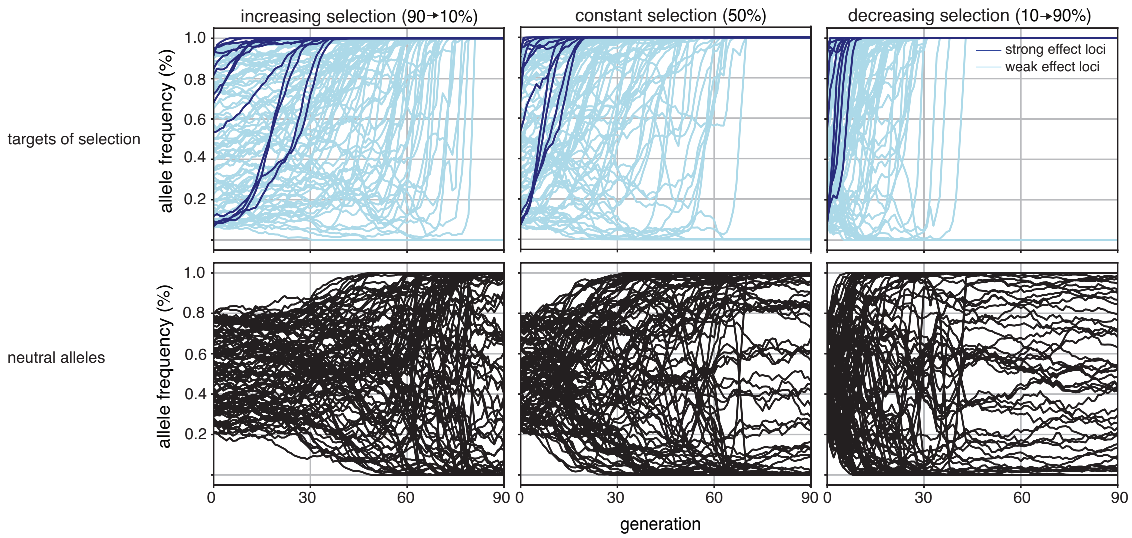

Figure 3: Trajectories of selected (blue) and not-selected alleles (black) for three different selection regimes (top). Strong effect loci (effect size >1) are shown in dark blue whereas weak effect loci (effect size ≤ 1) are shown in light blue. We also show the trajectories of 100 randomly picked neutral alleles that have starting frequency between 0.2 and 0.8. Data are shown for one replicate. Note that fixation of strong effect loci is delayed in the increasing regime (left) compared to the constant regime (middle) and the decreasing regime (right).

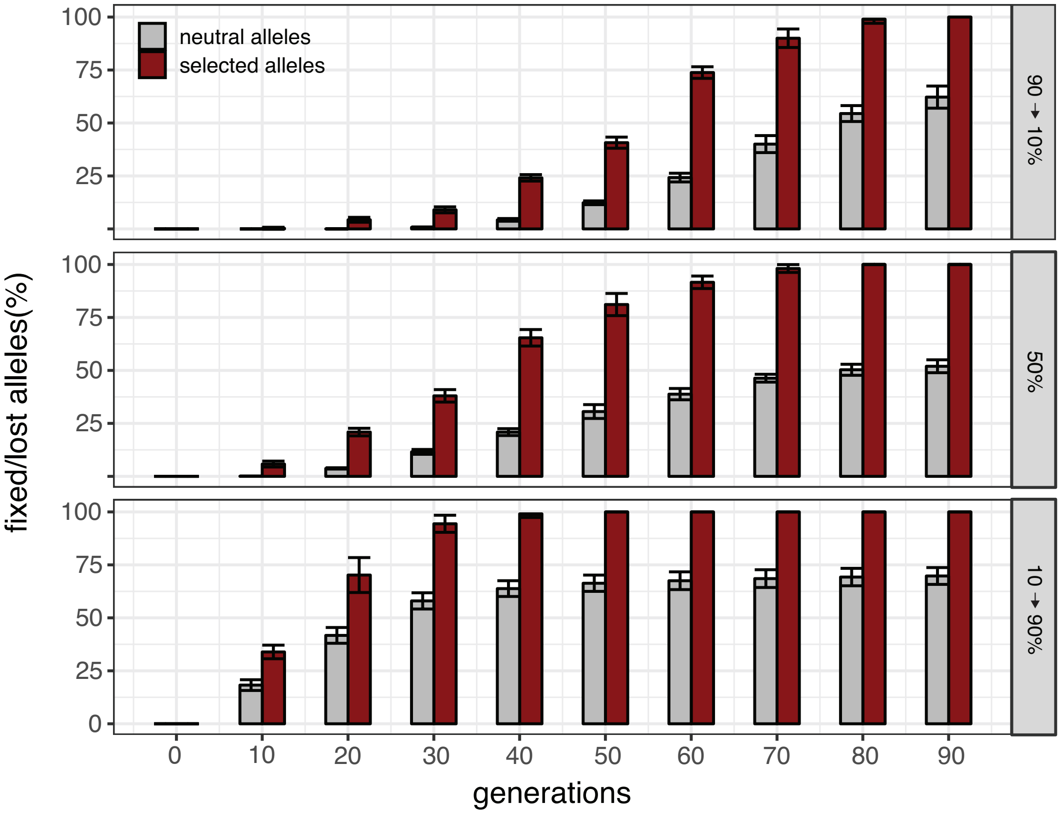

Figure 4: Fraction of fixed loci during an E&R studies with three different selection regimes. Results are shown separately for selected and neutral alleles. Lost selected alleles where quantified as fixed. The impact of an increasing (90 → 10%), a decreasing (10 → 90%) and a constant (50%) regime was evaluated. Error bars are based on 10 replicates. Results are solely shown for alleles with frequencies between 0.05 and 0.95 in the base population.

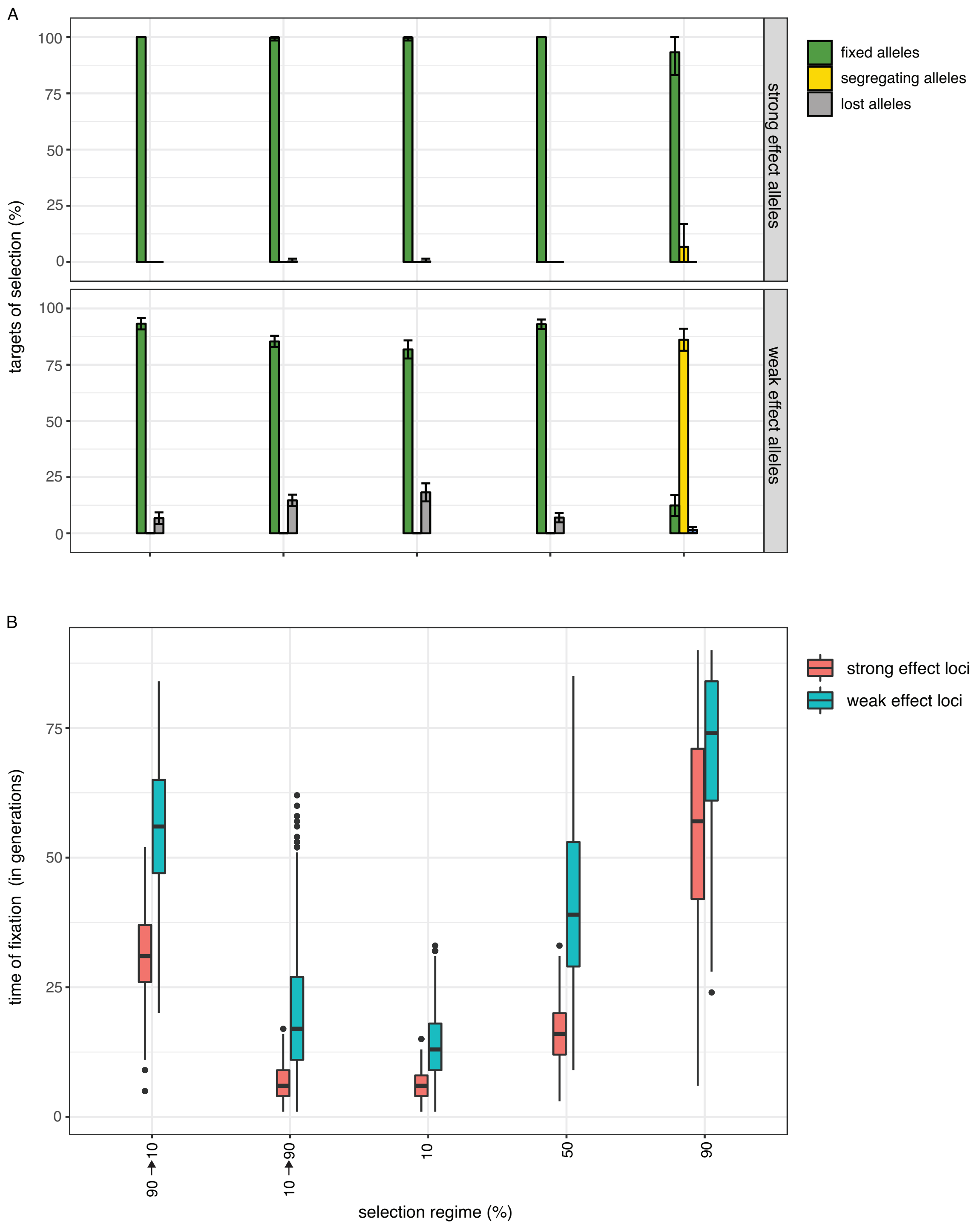

Figure 5: Fate of selected loci for 5 different selection regimes. The influence of an increasing ($90 \to 10\%$), a decreasing ($10 \to 90\%$) and three constant regimes (10%, 50%, 90%) was evaluated. A) Fraction of fixed (green), lost (grey, i.e. fixed in the wrong direction) and segregating loci (yellow) after 90 generations of selection. Results are shown for strong and weak effect loci. B) Time to fixation for strong and weak effect loci. Note that a linearly increasing regime delays fixation of selected loci compared to the constant regime with 50% selected individuals (Wilcoxon rank sum test; $p < 0.05$).

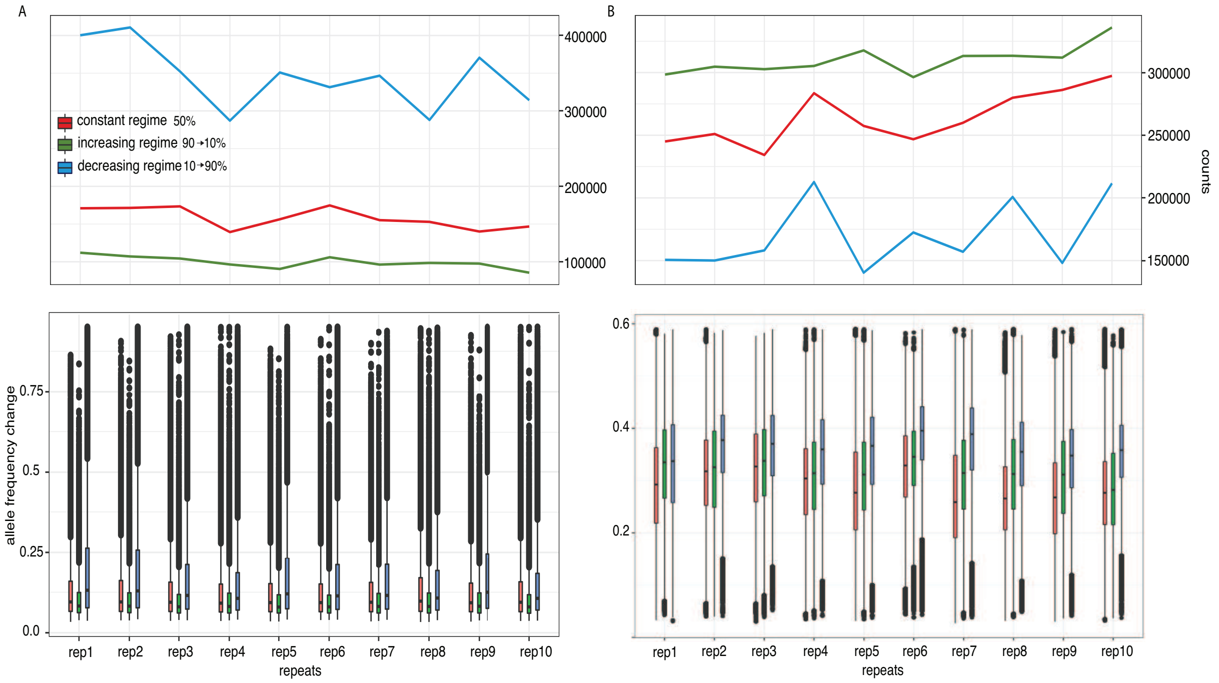

Figure 6: Overview of loci subject to hitchhiking (A) and drift (B) for an E&R study ($N = 1000$, 10 replicates, 90 generations) using an increasing, a constant and a decreasing regime. Results are shown for 10 different sets of selected loci ($rep1..rep10$). Loci were classified as subject to hitchhiking or drift if allele frequencies changed in the same direction in all 10 or in 4-6 replicates, respectively (excluding the selected loci). The number of loci subject to hitchhiking or drift is shown in the top panel and the allele frequency change of these loci in the bottom panel. Note that the increasing regime consistently generates fewer hitchhikers which also show a weaker response to selection than hitchhikers from the constant and decreasing regimes (Wilcoxon rank sum test; $p < 0.05$ in each repeat).

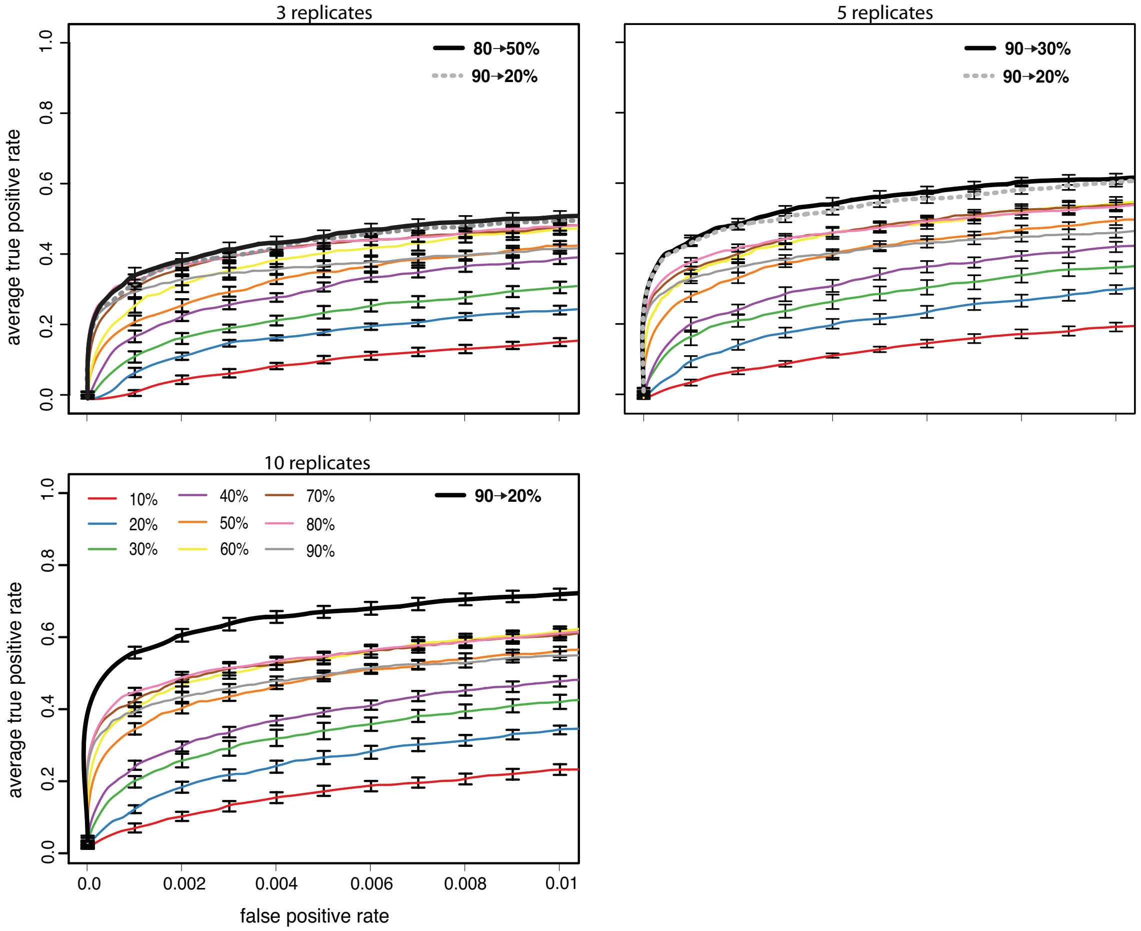

Figure 7: Influence of the number of replicates ($default = 10$). Although the best linearly increasing regimes consistently perform better than, or equal to, the best constant regimes, the advantage of linearly increasing regimes is most pronounced for 5 or more replicates. (Wilcoxon rank sum test with pAUC; $p_3 = 0.5$ for 4.9% increased performance; $p_5 = 0.0004$ for 16% increased performance; $p_{10} = 0.0002$ for 22.4% increased performance.)

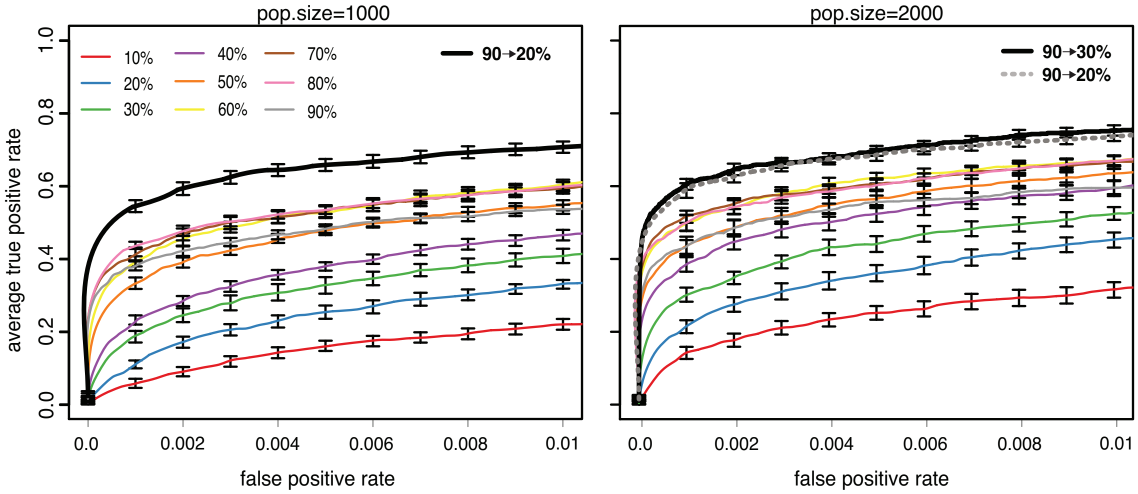

Figure 8: Influence of the population size ($default = 1000$). Linearly increasing selection regimes consistently perform better than the constant regimes (Wilcoxon rank sum test with pAUC; $p_{1000} = 0.0002$ for 22.4% increased performance; $p_{2000} = 1e - 05$ for 14.2% increased performance).

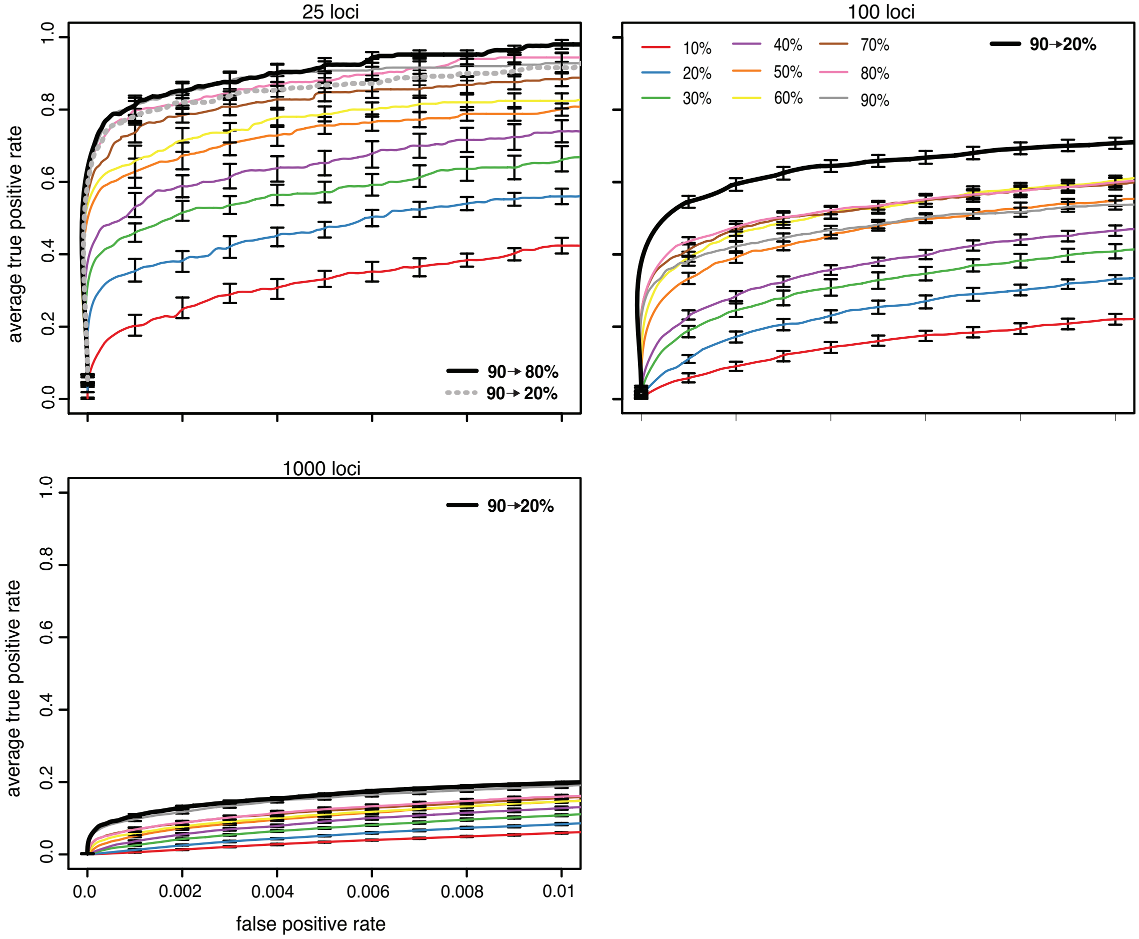

Figure 9: Influence of the number of QTNs ($default = 100$). Although the best linearly increasing selection regimes consistently perform better than, or equal to, the best constant regimes, the advantage of linearly increasing regimes is most pronounced for intermediate numbers of QTNs (Wilcoxon rank sum test with pAUC; $p_{25} = 0.73$ for 2% increased performance; $p_{100} = 0.0002$ for 22.4% increased performance; $p_{1000} = 0.14$ for 6% increased performance).

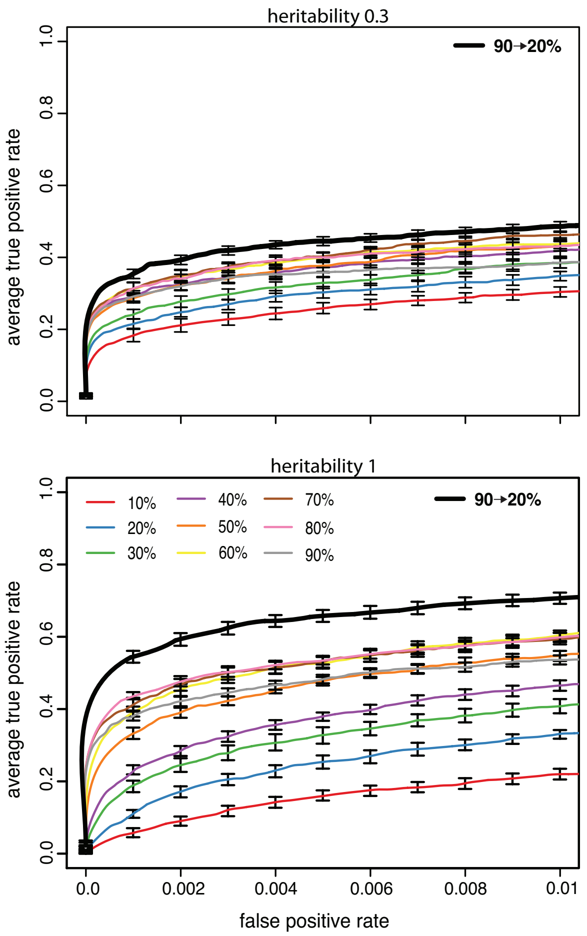

Figure 10: Influence of the heritability. The increasing selection regime consistently outperforms the constant selection regime, where the advantage is most pronounced for high heritabilities (Wilcoxon rank sum test with pAUC; $p_{0.3} = 0.035$ for 9% increased performance; $p_{0.6} = 0.035$ for 7.5% increased performance; $p_1 = 0.0002$ for 22.4% increased performance). Note that the influence of the selection regime diminishes with decreasing heritability (the ROC curves become more similar).

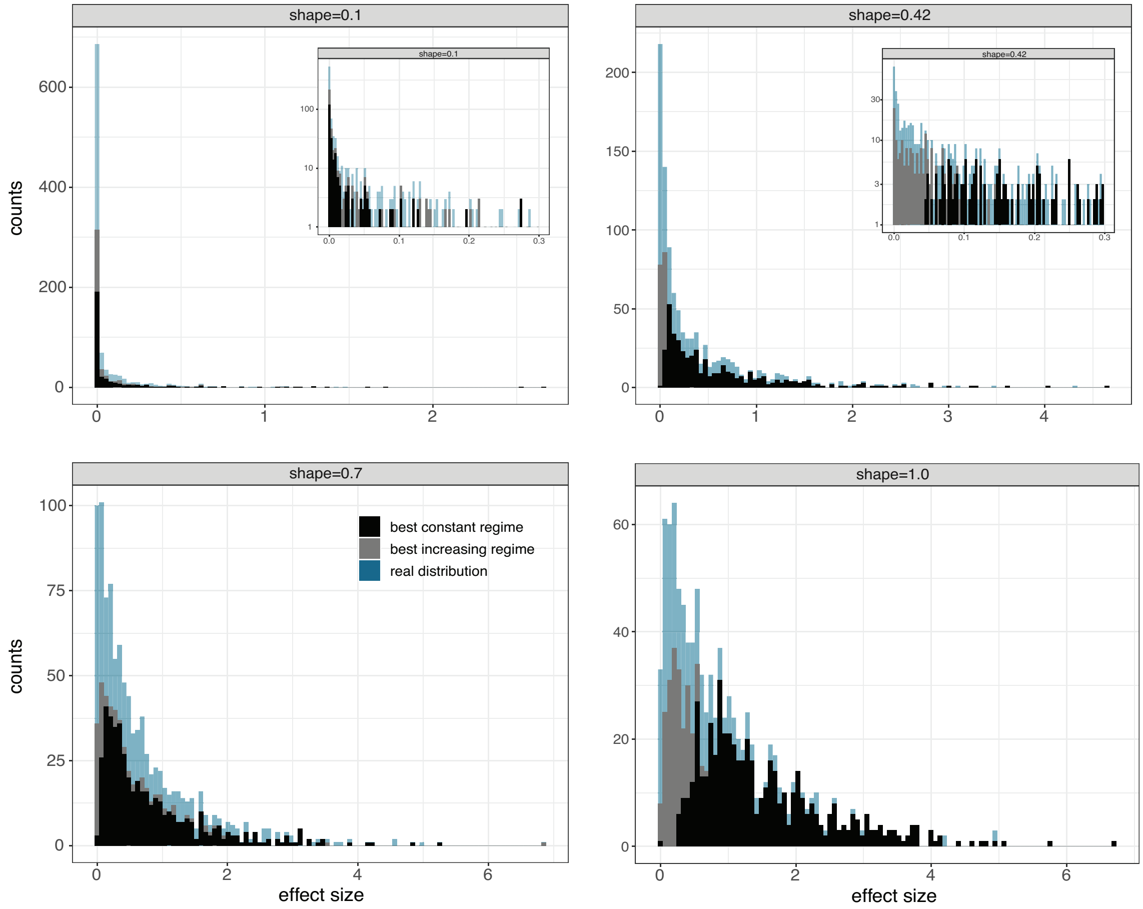

Figure 11: Histogram of the effect sizes of QTNs recovered with the best increasing (grey) and the best constant (black) regime compared to all simulated QTNs (blue). Results are shown for different gamma distributed effect sizes (top panel). Only QTNs among the 2,000 most significant SNPs were considered for this analysis. Results show the sum over 10 experiments with different random sets of QTNs. Inlays show the common logarithm of the counts for effect sizes ≤ 0.3. Note that the best increasing regime enables us to recover the effect size distribution more accurately than the best constant regime.

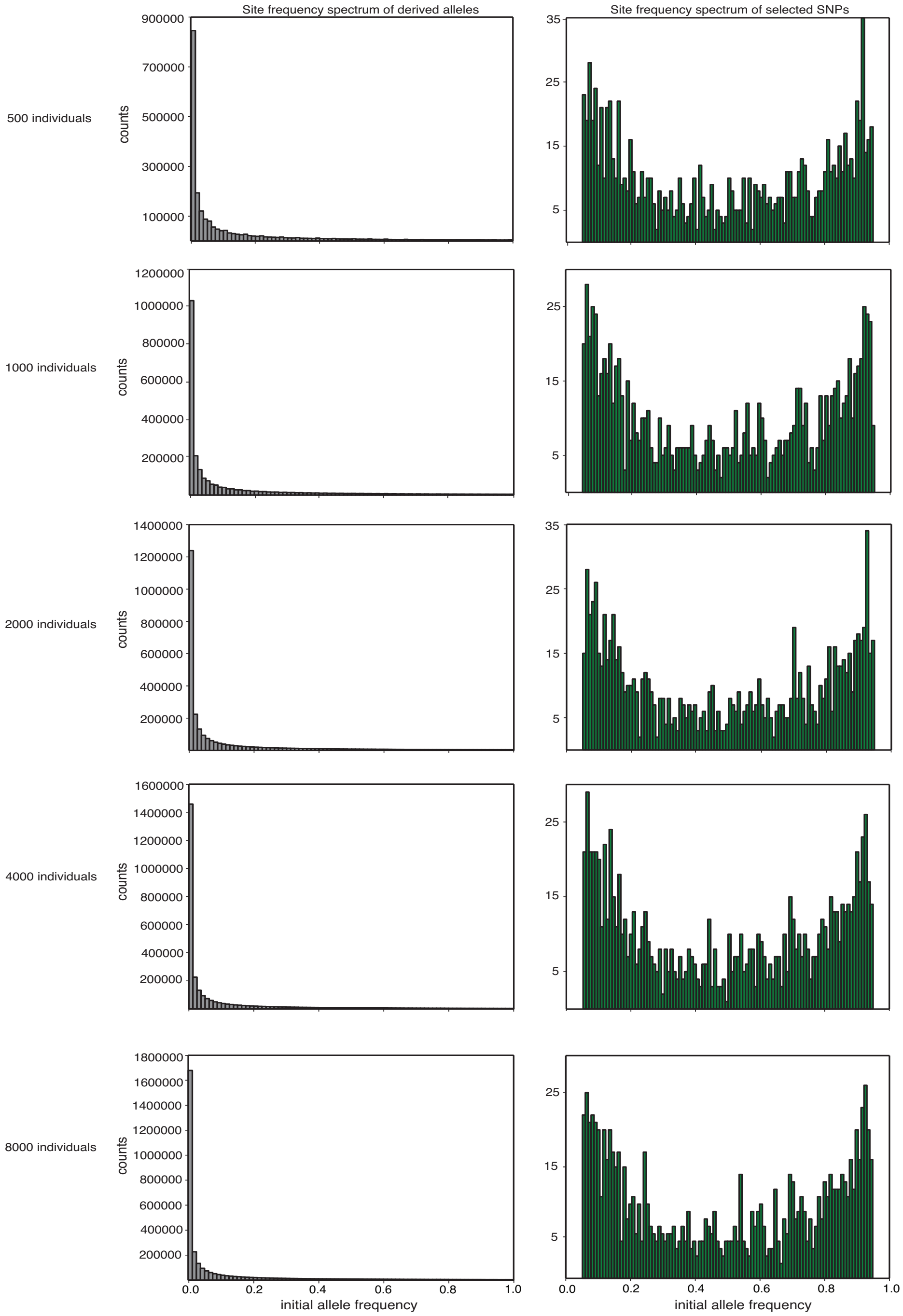

Figure 12: Site frequency spectrum of segregating SNPs (left column) and of the selected QTNs (right column). Results are shown for the five different population sizes used in the simulated GWAS. Note that segregating SNPs were polarized by the derived allele whereas QTNs were polarized by the sign of the effect size ($+a$).

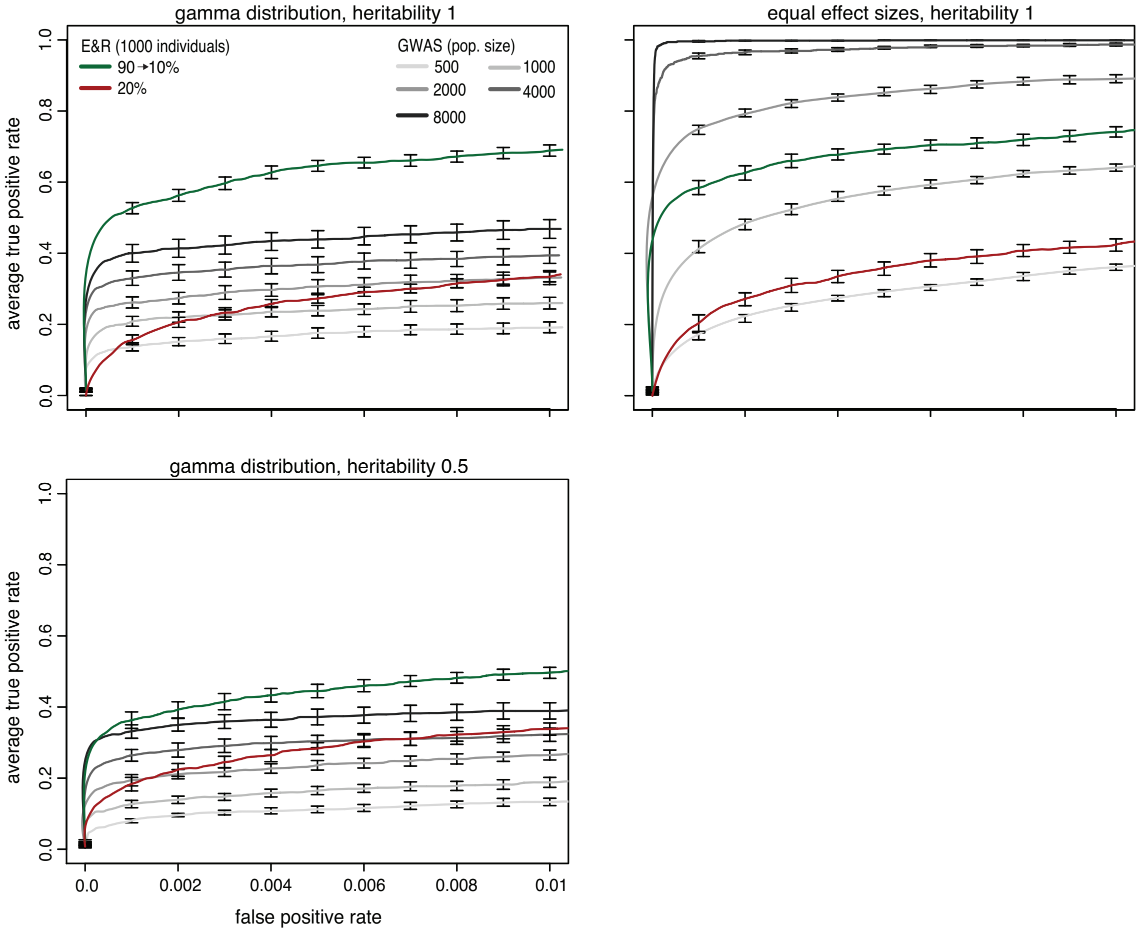

Figure 13: Influence of the heritability and effect size distribution on the performance of E&R and GWA studies. An E&R study with an increasing regime outperforms GWAS when the trait has a low heritability but not when effect sizes of QTNs are identical (Wilcoxon rank sum test with pAUC; $E\&R_{1000}$ vs $GWAS_{8000}$; with heritability 0.5: $p = 0.023$, with equal effect size: $p = 1.08e-05$).

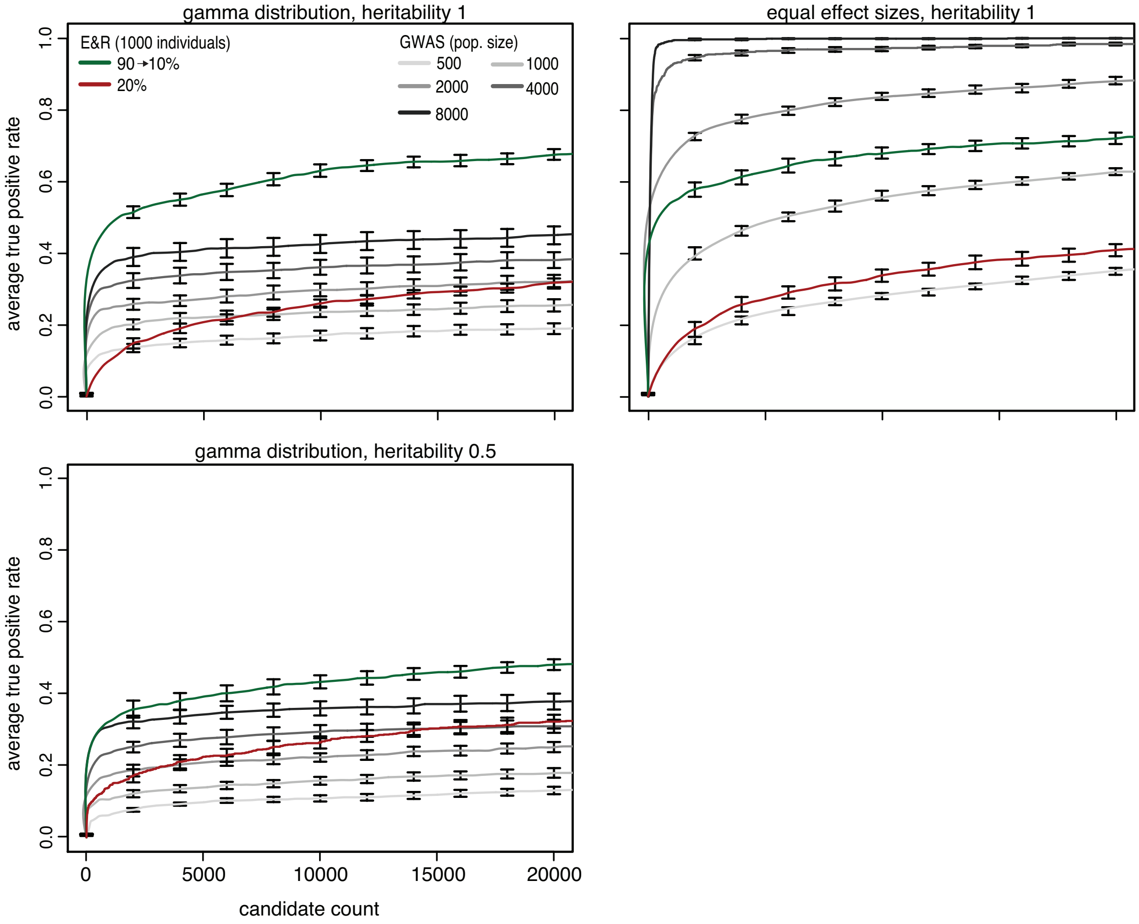

Figure 14: Influence of the heritability and effect size distribution on the performance of E&R and GWAS. The top 20,000 candidate loci are shown on the abscissa.

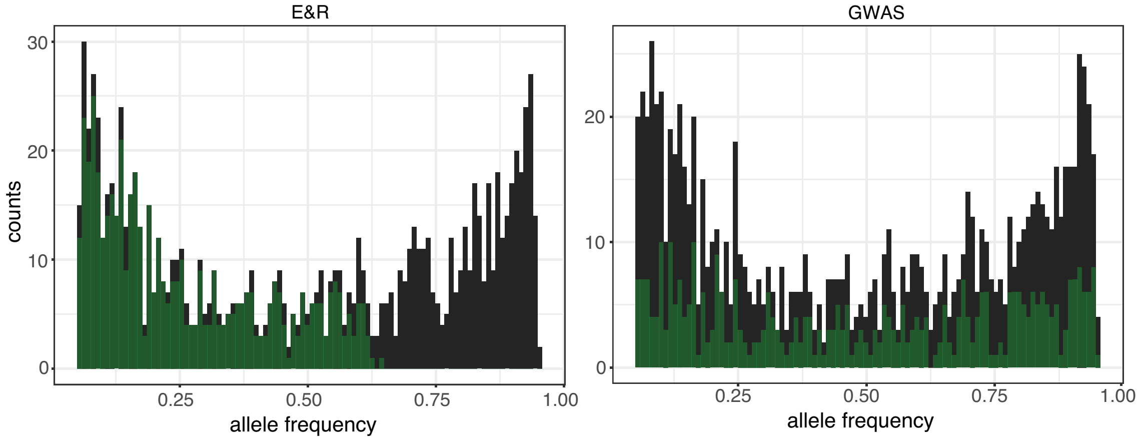

Figure 15: Starting allele frequency of QTNs identified with GWAS and E&R studies (green) compared to expectations (black). Only QTNs among the 2,000 most significant SNPs were considered for this analysis.

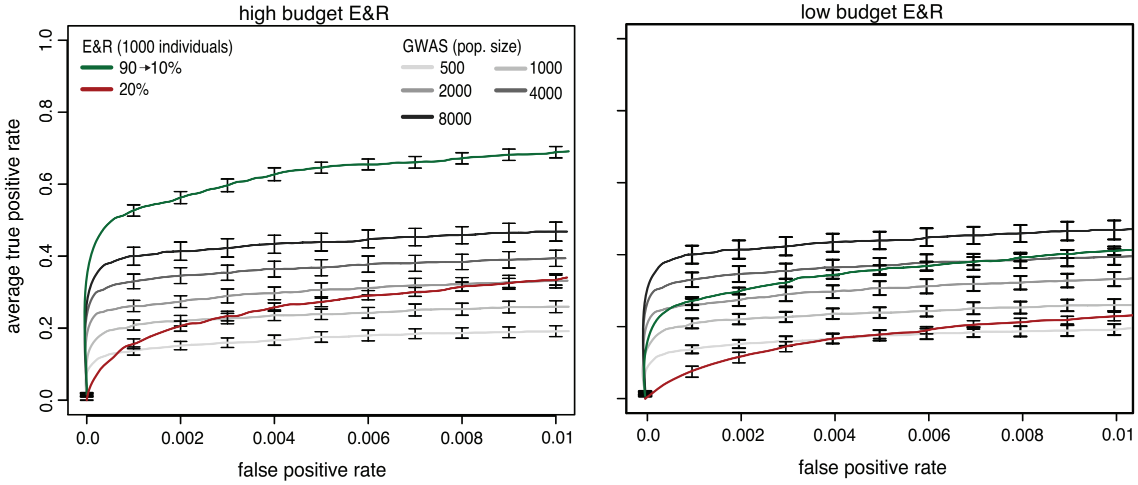

Figure 16: Performance of a low-budget E&R study ($N = 500$, 5 replicates, 45 generations), a high-budget E&R study ($N = 1000$, 10 replicates, 90 generations) and multiple GWAS with different populations sizes. A low-budget E&R study, with an improved selection regime has a similar performance to a GWAS with $1500 - 2000$ individuals.

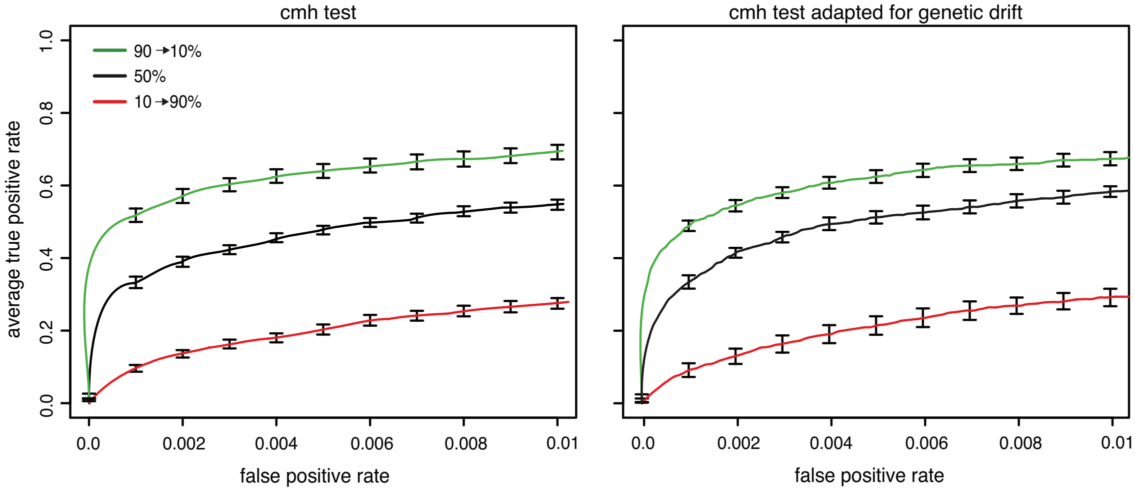

Figure 17: Performance of the classic cmh-test and a time-series based cmh-test adapted to E&R studies (Spitzer et al., 2019). For the adapted cmh-test we used allele frequency estimates at each $10^t h$ generation (10 time points in total with 90 generations of selection) and estimates of N_e based on the harmonic mean of the number of selected individuals (constant regime N_e=500; increasing/decreasing regime N_e=360). With the adapted cmh-test the increasing regime performed better than the constant regime (Wilcoxon rank-sum test with pAUC; $90 \rightarrow 10\%$ vs 50%; $p = 0.0002$) and the influence of the selection regime on the performance was significant (Kruskal–Wallis rank-sum test with pAUC; $p = 4.59e - 06$).

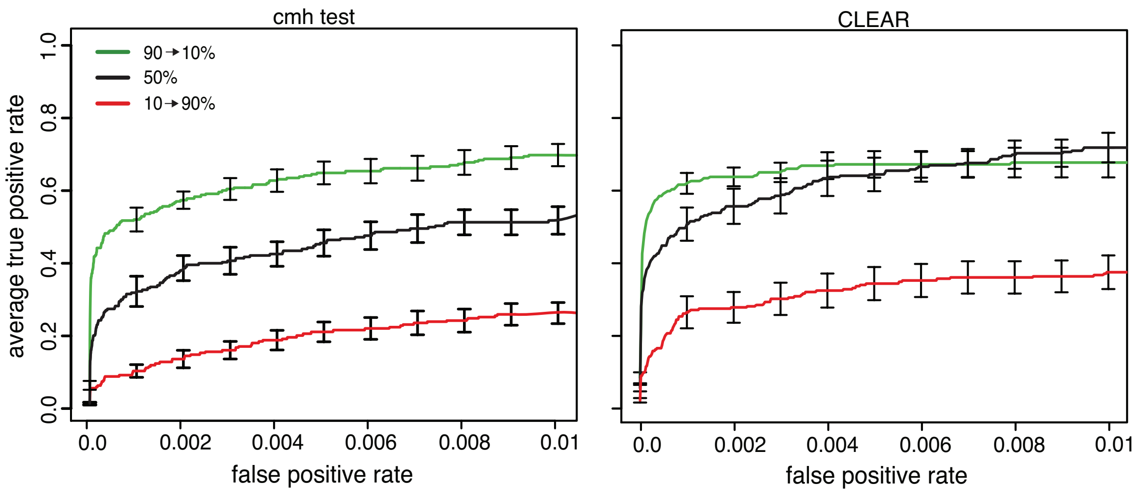

Figure 18: Performance of the cmh-test and of CLEAR, a time-series based approach for identifying selected loci in E&R studies (Iranmehr et al., 2017). We used allele frequency estimates of each $10^t h$ generation (10 time points in total with 90 generations of selection). Since CLEAR is computationally demanding we analyzed the data solely for one chromosome arm. The selection regime had a significant influence on the power to identify QTNs (Kruskal–Wallis rank-sum test with pAUC=0.01; $p = 5.79e - 05$). Furthermore, the increasing regime had a better performance than the constant regime (Wilcoxon rank sum test with pAUC=0.002; $90 \rightarrow 10\%$ vs 50%; $p = 0.035$).

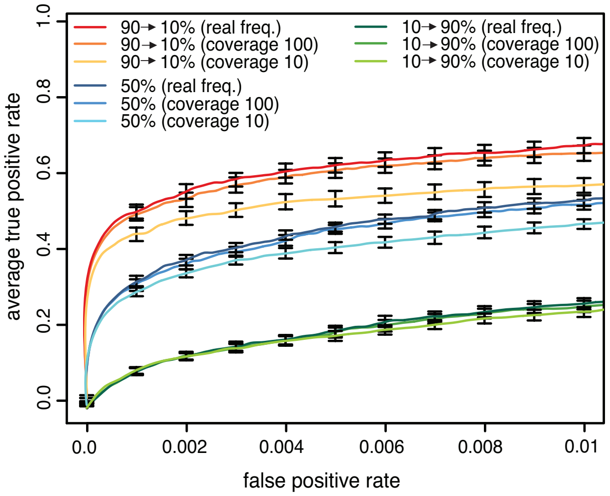

Figure 19: Influence of the coverage of Pool-Seq data on the power to identify QTNs. For each selection regime we show the performance using the actual allele frequency and allele frequency estimates obtained with Pool-Seq . Binomial sampling was used to model Pool-Seq with coverages 100 and 10. As expected the performance of E&R studies decreases with the coverage (Kofler and Schlötterer, 2014). Nevertheless the increasing regime outperformed the constant regime irrespective of the coverage (Wilcoxon rank sum test with pAUC; $90 \rightarrow 10\%_{realfreq}$ vs $50\%_{realfreq}$; $p = 2.165e{-}05$; $90 \rightarrow 10\%_{cov.100}$ vs $50\%_{cov.100}$; $p = 2.165e{-}05$; $90 \rightarrow 10\%_{cov.10}$ vs $50\%_{cov.10}$; $p = 2.165e - 05$).

Table 1: Overview of the three best selection regimes for different experimental designs and trait architectures. Selection regimes were ranked based on the partial area under the ROC curve ($pAUC$). Starting from the default conditions (top) we varied one parameter at a time (bold). g number of generations, r replicates, n number of loci, h^2 heritability, N population size, ga. gamma distributed effect sizes , eq. equal effect sizes

parameters	best regimes	pAUC±sd
g=90,r=10,n=100 h^2=1,N=1000,ga.	90 → 20%	0.00638 ± 0.0005
	90 → 30%	0.00633 ± 0.0004
	90 → 40%	0.00622 ± 0.0004
**g=20**,r=10,n=100,h^2=1,N=1000,ga.	90 → 70%	0.00362 ± 0.0004
	90 → 60%	0.00356 ± 0.0003
	80%	0.00352 ± 0.0003
g=90,**r=5**,n=100,h^2=1,N=1000,ga.	90 → 30%	0.00540 ± 0.0004
	90 → 40%	0.00529 ± 0.0004
	90 → 20%	0.00524 ± 0.0003
g=90,r=10,**n=1000**,h^2=1,N=1000,ga.	90 → 20%	0.00157 ± 0.0001
	90 → 10%	0.00154 ± 0.0001
	90 → 30%	0.00153 ± 0.0001
g=90,r=10,n=100,**h^2=0.6**,N=1000,ga.	90 → 10%	0.00450 ± 0.0003
	90 → 40%	0.00449 ± 0.0003
	90 → 20%	0.00448 ± 0.0004
g=90,r=10,n=100,h^2=1,**N=2000**,ga.	90 → 30%	0.00682± 0.0004
	90 → 10%	0.00670 ± 0.0005
	90 → 20%	0.00669 ± 0.0004
g=90,r=10,n=100,h^2=1,N=1000,**eq.**	90%	0.00915 ± 0.0002
	90 → 80%	0.00828 ± 0.0005
	90 → 70%	0.00759 ± 0.0002

Table 2: Performance of the 90 $\rightarrow$ 20% selection regime relative to the regime with the best performance with a given set of parameters. Experimental variations in the default parameters are in bold. We used pAUC to assess the performance of a regime and compute the ratio between the 90 $\rightarrow$ 20% increasing regime and the best regime (br). A value of 74%, for example, indicates that the 90 $\rightarrow$ 20% increasing regime is 26% less powerful than the best regime. g number of generations, r replicates, n number of loci, h^2 heritability, p population size, ga. gamma distributed effect sizes with shape 0.42, $ga_{0.1}$ gamma distributed effect sizes with shape 0.1, $ga_{0.7}$ gamma distributed effect sizes with shape 0.7, $ga_{1.0}$ gamma distributed effect sizes with shape 1.0, eq. equal effect sizes

parameters	best regime	$pAUC_{90\rightarrow 20}/pAUC_{br}$ (%)
**g=90**,r=10,n=100 h^2=1,p=1000,ga.	90 $\rightarrow$ 20%	100
**g=45**,r=10,n=100,h^2=1,p=1000,ga.	80 $\rightarrow$ 10%	99
**g=20**,r=10,n=100,h^2=1,p=1000,ga.	90 $\rightarrow$ 70%	85
g=90,**r=5**,n=100,h^2=1,p=1000,ga.	90 $\rightarrow$ 30%	97
g=90,**r=3**,n=100,h^2=1,p=1000,ga.	80 $\rightarrow$ 50%	97
g=90,r=10,**n=1000**,h^2=1,p=1000,ga.	90 $\rightarrow$ 20%	100
g=90,r=10,**n=25**,h^2=1,p=1000,ga.	90 $\rightarrow$ 20%	94
g=90,r=10,n=100,**h^2=0.6**,p=1000,ga.	90 $\rightarrow$ 10%	99
g=90,r=10,n=100,**h^2=0.3**,p=1000,ga.	90 $\rightarrow$ 20%	100
g=90,r=10,n=100,h^2=1,**p=2000**,ga.	90 $\rightarrow$ 30%	98
g=90,r=10,n=100,h^2=1,p=1000,**$ga_{0.1}$**	90 $\rightarrow$ 20%	100
g=90,r=10,n=100,h^2=1,p=1000,**$ga_{0.7}$**	90 $\rightarrow$ 30%	97
g=90,r=10,n=100,h^2=1,p=1000,**$ga_{1.0}$**	90 $\rightarrow$ 40%	97
g=90,r=10,n=100,h^2=1,p=1000,**eq.**	90%	74

References

H. Bastide, A. Betancourt, V. Nolte, R. Tobler, P. Stöbe, A. Futschik, and C. Schlötterer. A Genome-Wide, Fine-Scale Map of Natural Pigmentation Variation in *Drosophila melanogaster*. *PLoS Genetics*, 9: e1003534, 2013.

J. Comeron, R. Ratnappan, and S. Bailin. The Many Landscapes of Recombination in *Drosophila melanogaster*. *PLoS Genetics*, 8(10):e1002905, 2012.

A. Iranmehr, A. Akbari, C. Schlötterer, and V. Bafna. CLEAR: Composition of likelihoods for evolve and resequence experiments. *Genetics*, 206(2):1011–1023, 2017.

R. Kofler and C. Schlötterer. A Guide for the Design of Evolve and Resequencing Studies. *Molecular biology and evolution*, 31(2):474–483, 2014.

K. Spitzer, M. Pelizzola, and A. Futschik. Modifying the Chi-square and the CMH test for population genetic inference: adapting to over-dispersion. 2019. URL `http://arxiv.org/abs/1902.08127`.
